# Development of spontaneous neuropathy in NF-κBp50-deficient mice by calcineurin-signal involving impaired NF-κB activation

**Published:** 2011-08-11

**Authors:** Tomoko Nakamura-Yanagidaira, Yasuko Takahashi, Kenji Sano, Toshinori Murata, Takuma Hayashi

**Affiliations:** 1 Department of Ophthalmology, Shinshu University Graduate School of Medicine, Nagano, Japan; 2 Department of Laboratory Medicine, Shinshu University Hospital; 3 Department of Immunology and Infectious Disease, Shinshu University Graduate School of Medicine, Nagano, Japan

## Abstract

**Purpose:**

The transcriptional regulator, nuclear factor-kappa B (NF-κB)/Rel family are involved in neuronal cell death and survival. Previously, we reported that NF-κBp50-deficient (p50-deficient) mice exhibit many features resembling human normal tension glaucoma (NTG). The developmental mechanism of human NTG is not clearly understood, and a radical curative treatment has yet to be established. Our aim is to elucidate the signal cascade which mediates the spontaneous optic neuropathy in p50-deficient mice as a model of NTG.

**Methods:**

To demonstrate the expression and activation of pro-apoptotic factors, which mediate the death of retinal ganglion cells (RGCs) in p50-deficient mice, western blot (WB) and luciferase reporter assays with retinas from p50-deficient and wild type mice, and cultured RGC-5 cells were performed. Furthermore, we tested the neuroprotective effects of chemical reagents (memantine, lomerizine, and tacrolimus) against N-methyl-D-aspartate (NMDA)-susceptible RGC damage according to in vitro experiments with RGC-5 cells. To elucidate the NF-κB-mediated death signaling, the effects of chemical reagents on spontaneous optic neuropathy were examined by histopathological studies.

**Results:**

WB experiments and luciferase reporter assays showed that NF-κB-inducible BCL2-associated X protein (Bax) and a pro-apoptotic factor, activated caspase 3 were expressed in the retina of p50-deficient mice as well as NMDA-treated RGC-5 cells. Further, the constitutivelyactivecleaved forms of calcineurin (CaN), which havebeen reported to lead to apoptosis, were detected in the retina of p50-deficient mice as well as NMDA-treated RGC-5 cells. Pre-treatment with tacrolimus markedlyprotected RGC-5 cells from NMDA-induced neurotoxicity, and then both spontaneous RGC death and degenerative changes to the optic nerve in p50-deficient mice were significantlyreduced by the chronic administration of tacrolimus. The experiments with cultured RGC-5 cells supported the results of histological examinations with p50-deficient mice, suggesting that CaN activation leads to NF-κB-induced Bax activation and caspase 3 activation, and mediates spontaneous optic neuropathy in p50-deficient mice.

**Conclusions:**

Research findings show that the chronic administration of tacrolimus significantly reduces spontaneous optic neuropathy in p50-deficient mice. We demonstrated a potential CaN signal cascade, which spontaneously induces age-dependent RGC death and degenerative optic nerve changes in p50-deficient mice.

## Introduction

Glaucoma, one of the most common causes of visual impairment worldwide, is characterized by the apoptosis of retinal ganglion cells (RGCs) [1]. Although increased intraocular pressure (IOP) has long been considered the primary cause of the cell death, evidence from studies on normal tension glaucoma (NTG) suggests other factors to be involved in the apoptosis of RGCs, which is induced by a potential neurotoxic role for glutamate, genetic background, and autoimmunity [2-4]. Although most anti-glaucomatous reagents are used to lower IOP, in some cases, the patient's condition deteriorates in spite of an IOP within the normal range. Identifying factors, which are quite independent of IOP, would be necessary to understand the pathogenesis of glaucoma and guide efforts toward improved therapeutics.

Nuclear factor-kappa B (NF-κB), which acts as a transcription factor, plays a key role in cell survival or the death signaling pathway, acute central nervous system (CNS) trauma, and chronic neurodegenerative disorders [5,6].The NF-κB family, whichis mainlycomposed of p50/p65(RelA) heterodimers, is found in almost all animal cell types, and is involved in cellular responses to stimuli such as stress and cytokines [7]. In unstimulated cells, NF-κB is sequestered to the cytoplasm by a family of inhibitors called IκBs. With the degradation of IκB inhibitor, the NF-κB is then free to enter the nucleus, where it can turn on the expression of specific genes. Recent reports suggest that the binding site of the heterodimer p50-p65 could also be occupied by the homodimer p50-p50, whereupon p50-p50 may function as a repressor to regulate p50-p65's role as a transcription factor essential for neuronal response [7]. Impaired regulation of NF-κB has been linked to various diseases, such as cancer, inflammatory disorders, and autoimmune diseases, and has also been implicated in the processes of synaptic plasticity and memory [8]. In the CNS, it has been reported that the activated NF-κBp65(RelA) may participate in glutamate-induced neurotoxicity, N-methyl-D-aspartate (NMDA)-induced retinal neuronal cell death,retinal ischemia, and reperfusion injury [9-12]. However, the precise role of NF-κB in cell death within the CNS is controversial.

In many types of neuron, this excitotoxicity appears to be mediated predominantly by signaling pathways, including Ca^2+^ influx through the NMDA receptor, a subtype of the glutamate receptor [13]. For these reasons, it is likely that NMDA antagonists, memantine, dizocilpine (MK-801), and Ca^2+^ channel blockers, such as flunarizine, verapamil, nicardipine, and lomerizine, prevent retinal damage caused by NMDA [14-16]. Calcineurin (CaN) is a Ca^2+^-calmodulin-dependent phosphatase highly expressed in the CNS and retina [17,18]. Activation of CaN leads to apoptosis of cultured neurons [19]. Tacrolimus, a CaN inhibitor, exhibits a neuroprotective effect in a wide range of models of acute apoptotic neuronal death in the CNS, trauma,and stroke [20,21]. In the retina, tacrolimus has been reported to confer neuroprotection on RGCs after optic nerve crush [22]. In addition, an increased IOPis associated with the CaN-mediated mitochondrial apoptotic pathway [23]. Furthermore, tacrolimus has been shown to inhibit glutamate-induced apoptosis in cultured RGCs [24]. Although the neuroprotective effects of these chemical reagents have been examined in transient studies employing glaucoma models, it has yet to be demonstrated whether the chronic administration of chemical reagents to NTG-animal models has protective effects against neuropathy.

Previously, we reported that NF-κBp50-deficient (p50-deficient) mice exhibit a spontaneous loss of RGCs independent of IOP, characteristic excavation of the optic nerve head, and degenerative changes to the optic nerve, with manyfeatures resembling human NTG [25]. However, the precise mechanism involved is still unknown. In this study, we tried to identify the death signaling cascade in the initiation of spontaneous optic neuropathy in p50-deficient mice as a model of NTG. The experiments demonstrated neuroprotective effects of several chemical reagents, especially tacrolimus, against NMDA-induced neurotoxicity and spontaneous RGC death in p50-deficient mice. The defect in the p50/p50 homodimer, which functions as a repressor to regulate p50-p65's role, results in optic neuropathy via a CaN signal cascade involving impaired NF-κB activation in p50-deficient mice.

We provide a new insight into the role of NF-κBp50 in the pathophysiologyof optic neuropathy, and present experiments involving p50-deficient mice demonstrating the CaN-signaling molecules including NF-κB, suggesting targets for the development of therapeutic reagents. Our research findings mayprovide new targets for therapeutic intervention in human NTG.

## Methods

### Animals

We purchased mice homozygous for the Nfkb^1tmBal^ target mutation, which have a B6/129-F2 genetic background, from Jackson Laboratories (Bar Harbor, ME), and C57BL/6J mice from CLEAJapan, Inc. (Tokyo, Japan). All wild type, homozygous mice deficient in p50 were littermates of F8 heterozygous mice generated by backcrossing with C57BL/6J mice. All animal-related procedures were performed in compliance with the National Institute of Health Guide for the Care and Use of LaboratoryAnimals and theARVO Statement for the Use ofAnimals in Ophthalmic and Vision Research. These mice were kept in a specific pathogen-free environment at Shinshu University's animal facilities, in accordance with institutional guidelines (approval no. 03–28–008). A total of 280 mice (wild type mice; n=90, p50-deficient mice; n=190, at 3 weeks to 10 months of age) were used in this study.

### Labeling and counting in RGCs

Retrograde labeling was performed as reported [25]. Mice were anesthetized by the intraperitoneal administration of Nembutal (50 mg/kg; Dainippon Sumitomo Pharma, Osaka, Japan). The animals were placed in a stereotaxic apparatus (Narishige, Tokyo, Japan) and the scalp was incised at the midline and retracted laterally to make a burr hole at an appropriate location (2.0 mm posterior to the bregma, 1.2 mm left of the midline) determined according to the mouse brain map. A window was drilled in the scalp, the neurotracer dye Fluoro-Gold (a 4% solution in saline; Fluorochrome, Denver, CO) was applied (1 μl at a rate of 0.5 μl/min in each hemisphere) using a Hamilton syringe, and the skin over the wound was sutured. Five days after the application of Fluoro-Gold, mice were euthanized and, after their eyes were enucleated, the retinas were detached as flattened whole mounts in 4% paraformaldehyde in PBS. Labeled cells from 10 fields of identical size (230×150 μm) were counted under a fluorescence microscope. The counted fields were located at approximately the same distance from the orra serata (500 μm). Fields were counted by 2 blinded observers, and the average number of RGCs per field was calculated for each retina and analyzed by ANOVA with Bonferroni correction.

### Immunohistochemistry (IHC) and immunofluorescence

The retinas of mice ranging in age from 3 weeks to 10 months were used for research experiments. Mice were euthanatized and their eyes were immediately enucleated and fixed (4% paraformaldehyde). Paraffin-embedded retinas were used for IHC and immuno-fluorescence experiments. The sections were dewaxed and rinsed with phosphate-buffered saline (PBS). Then, they were incubated with 10% BSA (BSA) for 120 min at room temperature. After rinsing with PBS, the sections were incubated with primary antibodies, rabbit polyclonal antibodyto p65 (1:200, Santa Cruz Biotechnology, CA), anti-caspase 3 rabbit polyclonal antibody (1:100; EMD Bioscience, Darmstadt, Germany), and Bax 6A7 monoclonal antibody (1:200; TREVIGEN, Gaithersburg, MD), overnight at 4 °C. Normal rabbit or mouse antiserum was also used as a negative control. After a wash with PBS, the secondaryantibody, Alexa Fluor 488-conjugated donkey anti-rabbit IgG (1:200, Invitrogen Corp., Carlsbad, CA), Alexa Fluor 546-conjugated goat anti-rabbit antibody (1:200; Invitrogen), and Alexa fleur 488 conjugated mouse IgG1 antibody (1:200; Invitrogen) were reacted at room temperature for 2 h. Finally, the sections were coverslipped with antifade mounting medium (VECTASHILD; Vector Laboratory, Inc., Burlingame, CA). Sections were examined under a Leica laser scanning confocal microscope (TCS SP2 AOBS; Leica Microsystems GmbH, Wetzlar, Germany). For in situ TdT-mediated dUTP nick-end labeling (TUNEL) assays, paraffin-embedded retinas were used. The sections were dewaxed and rinsed with PBS. The TUNEL assay was performed using Apop Tag Fluorescein In situ Apoptosis Detection Kit according to the manufacturer's instructions (MILLIPORE, Billerica, MA). After labeling, the slides were mounted with mounting medium (VECTASHIELD; Vector Laboratories) for fluorescence microscopy with PI nuclear staining.

### Western blot

The retinas were dissected from mouse eyes derived from wild type and p50-deficient mice, and homogenized in modified RIPA buffer (50 mM Tris [pH7.4]; 1% NP-40; 0.25% Na-deoxycholic acid; 150 mM NaCl; 1 mM EDTA; 1 mM NaF; 1 mM PMSF; 1 mM Na_3_VO_4_; and aprotinin, leupeptin, pepsatain at 1 μg/ml each). The samples were heated at 95 °C for 10 min and centrifuged at 20,000× g for 20 min, and the supernatants were stored as retinal crude extracts at −30 °C. These extracts (10 μg) were run on a 10% SDS–PAGE, and an immunoblot analysis was performed with a rabbit monoclonal antibody to NF-κBp50 (Epitomics, Inc., Burlingame, CA), rabbit polyclonal antibody to NF-κBp65, rabbit polyclonal antibody to Calcineurin (Exalpha Biologicals Inc., Watertown, MA), rabbit polyclonal antibody to caspase 3 (EMD Bioscience), rabbit polyclonal antibody to p-actin (Santa Cruz technology, Inc., Santa Cruz, CA), and mouse monoclonal antibody to Bax (TREVIGEN) using standard procedures. Immunodetectable proteins were visualized and enhanced using the ECL western blot system (GE Healthcare Bio-Science, Uppsala, Sweden).

### Cytosol and nuclear extracts

Cultures of retinal ganglion cells (RGC-5, a rat ganglion cell line transformed using the E1A virus), which were provided by Dr. Neeraj Agarwal (UNT Health Science Center, Fort Worth, TX), were maintained in Dulbecco's modified Eagle's medium (DMEM; Sigma-Aldrich, St. Louis, MO) containing 10% fetal bovine serum (FBS; Valeant, Costa Mesa, CA), 100 U/ml penicillin (Meiji Seika Ltd., Tokyo, Japan), and 100 μg/ml streptomycin (Meiji Seika Ltd., Tokyo, Japan) in a humidified atmosphere of 95% air and 5% CO_2_ at 37 °C. The RGC-5 cells were passaged by trypsinization every 3–4 days. Cytosol extracts and nuclear extracts were prepared from 1×10^6^ RGC-5 cells. Cells were harvested, centrifuged for 10 min at 130× g, washed in 1 ml of ice-cold PBS, and centrifuged for 15 s at 17,000× g in an Eppendorf Brinkman-5412 centrifuge at 4 °C. Cells were pelleted and washed once in 0.4 ml of buffer A (10mM Hepes, pH7.8; 10 mM KCl; 2 mM MgCl2 ; 1 mM dithiothreitol [DTT]; 0.1 mM EDTA; 0.1 mM phenylmethylsulfonyl fluoride [PMSF]; 5 μg/ml antipain [Sigma, St Louis, MO], and 5 Ig/ml leupeptin [Sigma]) and incubated on ice for 15 min. Then, 25 μl of a 10% Nonidet P-40 solution (Sigma) was added, and cells were vigorously mixed for 15 s and centrifuged for 30 s at 17,000× g. Pelleted nuclei were resuspended in 40 μl of buffer C (50 mM Hepes, pH 7.8; 50 mM KCl; 300 mM NaCl; 0.1 mM EDTA; 1 mM DTT; and 0.1mM PMSF; 10% [v/v] glycerol), mixed for 20 min, and centrifuged for 5 min at 17,000× g at 4 °C. The supernatant containing the nuclear proteins was harvested, the protein concentration was determined, and the samples was stored at −80 °C.

### Cell transfections and reporter assays

RGC-5 cells were seeded at 70% confluence in 6-well plates, and a total of 2 μg of luciferase reporter vectors (Bax [full] promoter-Luc and Bax (ΔκB) promoter-Luc), provided by Dr. Chotteau-Lelievre (University of Sciences et Technologies, Cedex, France), was transfected into RGC-5 cells with FuGENE6 Transfection Reagent according to the manufacturer's recommendations (Roche Diagnostics, Indianapolis, IN). All transfections contained 200 ng of pCMV-LacZ (Roche Diagnostics) as an internal transfection efficiency control. Glutamate (final concentration, 50 μg) was added to the culture medium at 48 h after transfection and the cells were washed for the preparation of whole cell lysates. Cell lysates were prepared 24 h later for the assays of luciferase (Promega Corp., Madison, WI) and p-galactosidase (Galactolight; Roche Applied Science) activities followingthe manufacturer's instructions and using a GloMax®20/20 Luminometer (Promega Corp.). The measured luciferase activity was corrected by using co-transfected pCMV-p-galactosidase activity as an internal control.

### N-methyl-D aspartate (NMDA) injection and morphometric analysis

The intravitreal injection of NMDA was performed as described previously [25]. A total of 1 μl of 5 or 10 nM solution of NMDA in PBS was injected into the vitreous of one eye. PBS alone was injected into the other eye as a negative control. At 24 h after the injection, the eyes were enucleated and fixed in 4% paraformaldehyde in PBS (Wako, Tokyo, Japan), dehydrated, and embedded in paraffin. Transverse sections were made though the optic disc and stained with hemotoxylin and eosin. The surviving cells of 0.35 mm in length at 0.3 mm from the edge of the optic disc and orra serata in the ganglion cell layer (GCL) were counted, except the cells infiltrating the GCL. The average number of cells per field was analyzed using the Mann–Whitney U test.

### Chemical reagents

NMDA receptor antagonists, MK-801 and memantine, were purchased from Sigma-Aldrich Japan (Tokyo Japan). The T- and L-type calcium channel blockers and a potential anti-migraine drug, lomerizine hydrochloride, were supplied by Nippon Organon (Osaka, Japan). The calcineurin inhibitor tacrolimus was supplied by Astellas Phama Inc. (Tokyo, Japan). Lomerizine was dissolved in 2% dimethyl sulfoxide (DMSO) containing 0.2% tartaric acid. MK-801 and memantine were dissolved with distilled water, and tacrolimus was dissolved with saline. The p50-deficient mice and wild-type mice aged 2 months were daily pre-treated intraperitoneally with memantine (10 mg/kg), MK-801 (0.5 mg/kg), lomerizine (1 mg/kg), or tacrolimus (2, 0.5 and 0.2 mg/kg) for one week before the NMDA injection (shown in Appendix 1-Figure 3). These mice were given an intravitreous injection of 5 nM NMDA, which is a relatively low concentration for causing neurotoxicity.

### Transfection and cell death assay

A total of 2 μg of NF-κBp50 expression vector, provided by the NIH AIDS Research and Reference Reagent Program (Germantown, MD), was transfected into RGC-5 cells with FuGENE6 Transfection Reagent according to the manufacturer's recommendations (Roche). All transfections contained 200 ng of pCMV-LacZ (Roche Diagnostics) as an internal control. The transfected-cells were cultured with the culture medium containing glutamate (final concentration, 50 μM) or N-methyl D aspartate (NMDA; final concentration, 50 nM) for 24 h after the transfection and washed for the preparation of whole cell lysates as well as nuclear and cytosolic extracts. The RGC-5 cells were pre-treated with tacrolimus (final concentration, 50 μM or 100 μM) for 24 h, and then glutamate or NMDA was added for 24 h. After stimulation with glutamate or NMDA, RGC-5 cells were treated with Tripsin-EDTA solution (GIBCO, Grand Island, NY) and scraped from the plate. Viable cells were counted by trypan blue exclusion. The average number of live RGC-5 cells was analyzed to obtain the mean values±SD of live cells.

### Oligonucleotides and electrophoretic mobility shift assay (EMSA)

Double-stranded oligodeoxynucleotides were prepared by the phosphoramidate method with a DNAsynthesizer and purified on an OPC cartridge (FASMAC, Inc. Kanagawa, Japan). They correspond to wild-type κB (5′-GAT CTA GGG ACT TTC CGC TGG GGA CTT TCC AG-3′) binding motifs of the human immunodeficiency virus type 1 (HIV-1κB) enhancer or the SP1-binding motif (5′-ATT CGA TCG GGG CGG GGC GAG C-3′). For EMSA, nuclear extracts were incubated for 30 min at 37 °C in a total volume of 10 μl containing 10 mM Hepes-NaOH (pH 7.9), 50 mM KCl, 5 mM Tris-HCl (pH 7.0), 1mM DTT, 15 mM EDTA, 10% glycerol, 1.0 μg of poly (dI'dC), and 4 ng of wild-type HIV-1κB or SP1 oligonucleotides. The resulting DNA–protein complexes were resolved by electrophoresis on nondenaturing 8% polyacrylamide gels with 0.5× Tris borate-EDTA buffer at 4 °C. NF-κB-binding activities wereexamined by EMSA as described [26].

### Chronic administration of various chemical reagents

In the chronic administration experiments, daily intraperitoneal injections of lomerizine (1 mg/kg), memantine (10 mg/kg), tacrolimus (2 mg/kg), or vehicle (PBS) to p50-deficient mice were initiated from the age of 2 months for 8 months (shown in Appendix 1-Figure 4). To elucidate the neuroprotective effects of several chemical reagents, RGC survival in control mice and mice of all treatment groups was examined by the retrograde fluorescence labelingof RGCs.

### Azan Mallory stain

Mice were euthanized and their eyes were immediately enucleated and fixed (4% paraformaldehyde). Paraffin-embedded retinas were used. The sections were dewaxed and rinsed with water. They were incubated in mordant containing potassium bichromate and trichloroacetic acid at room temperature for 10 min. After being rinsed with water, the sections were incubated in Orange G solution in a moisture chamber for 10 min at 4 °C. Following more rinsing in water, they were incubated in Azocarmine G solution in the moisture chamber again for 20 min at 4 °C, rinsed with water, and incubated in phosphotungustic acid and phosphomolybdic acid solution in the moisture chamber for a further 10 min at 4 °C. Following further rinsing with water, the sections were incubated in Aniline Blue solution in the moisture chamber for a final 15 min at 4 °C. After being rinsed with water, the sections were incubated using an ascending alcohol series.

### Morphometry and transmission electron microscopy

After anesthesia, transcardinal perfusion was performed with 4% paraformaldehyde, and the optic nerves were dissected starting at 2 mm behind the globe, and placed in fixative (approximately 20 ml of 2.0% glutaraldehyde and 2.0% paraformaldehyde in 0.1 M cacodylate buffer) for 12 h. The optic nerves were then post-fixed in 1% osmium tetroxide, dehydrated in ethanol, and embedded in EPOK812 (Okenshoji, Tokyo, Japan). For transmission electron microscopy, ultrathin sections were cut perpendicular to the long axis of the optic nerves on an ultramicrotome, stained with uranyl acetate and lead citrate, and examined using a JEM1200EX transmission electron microscope (JEOL, Ltd., Tokyo, Japan). For light microscopic observation, toluidine blue staining was used. The numbers of axons in 10 fields of identical size (18×18 μm) were counted using Image J software (National Institute of Heath, MD), and the average number of axons was analyzed by ANOVA (ANOVA) with the Mann–Whitney U test.

## Results

### Age-dependent spontaneous RGC death due to apoptosis in NF-κBp50-deficient mice

**Figure 1:**
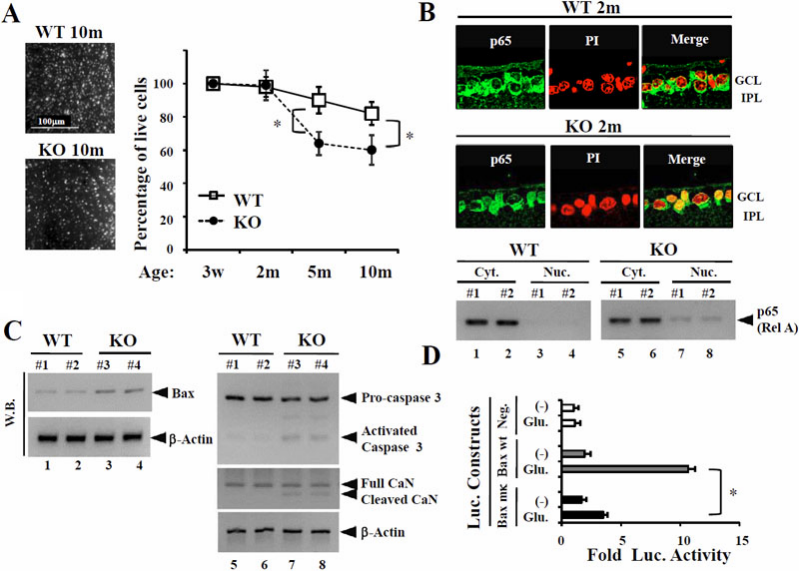
Activation of pro-apoptosis factors, caspase 3, Bax, and p65(RelA), and calcineurin in the retina of p50-deficient mice. **A**: Age-related ganglion cell death in p50-deficient (KO) mice. Retrogradely labeled cells from 10 fields of identical size (230×150 μm) in flat-mounted retinas were counted under a fluorescence microscope. The fields were located at approximately the same distance from the orra serata (500 μm). Scale bar=100 μm. The average number of RGCs per field was calculated for each retina and analyzed by Bonferroni correction. Data are the mean±SEM (each 8~12; *p<0.01). **B**: Immunohistochemical staining for NF-κBp65 expression at 2 months in wild-type (WT) mice and p50-deficient mice. NF-κBp65 translocates into the nucleus of RGCs in KO mice. GCL=ganglion cell layer, IPL=inner plexiform layer. Green; p65, Red; PI. Original magnification; 120×. Western blot analysis of NF-κBp65 and Bax protein levels in KO mice and WT mice. Immunoreactive bands of NF-κBp65 in the nucleus were slightly more intense in KO than WT mice. **C**: western blot analysis of pro-apoptosis factors, Bax, caspase 3 and p65(RelA), and calcineurin (CaN) levels in KO mice and WTmice. Bax protein levels were also slightly increased in KO mice. Activated caspase 3 and cleaved CaN were detected in KO mice. **D**: The luciferase reporter vectors containing the wild-type or mutated B sites of the PMBaxSacI *bax* promoter (−386 to −1) or the corresponding empty luciferase reporter vector (shown in Appendix 1-Figure 1) were transiently co-transfected with or without the NF-κBp50 expression vector in RGC-5 cells. RGC-5 cells, pre-treated with tacrolimus, were stimulated by glutamate for the final24 h, and then luciferase activities were measured. Values were normalized to those obtained with the co-transfected pSV-β-galactosidase expression vector. Each assay was performed at least three times in triplicate. Luciferase-reporter assays showed that glutamate-treatment markedly induced Bax expression due to NF-κB activation. Data are presented as the mean for three independent experiments (*S.D.).

NF-κB, which is expressed in RGCs as well as the CNS, plays important roles in such processes as synaptic activity and cell survival [6]. Morphometric evaluation indicated that at 5 months, there was no difference in the thickness and construction of each retinal layer, especially the inner nuclear layer (INL) and outer nuclear layer (ONL). The RGCs in p50-deficient mice at age 3 weeks, 2 months, 5 months, and 10 months were counted using a retrograde labeling method, and the number at 5 months and 10 months of age (n=each 8~12 mice) had markedly decreased compared with that in wild-type littermates (Figure 1A).

To demonstrate whether the p50-deficiency affected NF-κBp65 activation and RGC survival in the retina, we examined NF-κBp65 expression at 2 months of age. NF-κBp65 was detected in the cytoplasm of RGCs of wild-type mice. Unexpectedly, IHC clearly showed that NF-κBp65 spontaneously localizes in the nucleus of RGCs in p50-deficient mice under normal conditions (Figure 1B); the p50-deficiency in RGCs may result in the induction of NF-κBp65's neuronal toxicity. Western blot (WB) analysis strongly supports the IHC findings (Figure 1B). Bax and Caspase 3, which play a key role in the promotion of apoptosis, mediates stress-induced RGC death as well as glutamate-induced apoptosis in cultured neuronal cells [27-29]. Furthermore, Bax is required for the RGC death pathway in DBA/2J mice, which is an IOP-dependent glaucoma model. The WB results showed that Bax expression in the retinas of p50-deficient mice was slightly higher than that in wild-type mice, and further activated forms of caspase 3 were detected in the retinas from p50-deficient mice (Figure 1C). The constitutively active cleaved form of calcineurin (CaN) has been reported to lead to apoptosis by elevating IOP in experimental glaucoma [28]. The WB results showed the presence of the full-length CaN in all retinas examined, and that cleaved CaN occurred only in retinas from p50-deficient mice (Figure 1C).

To demonstrate whether glutamate treatment directly induces Bax expression via NF-κB activation, luciferase reporter assays using cultured RGC-5 cells were performed. Although Bax promoter activity was strongly induced in the experiment using the reporter construct containing the wild-type B-binding site after glutamate-treatment, the activitywas not clearly detected using the reporter construct containing the mutated B-binding site (Figure 1D, Appendix 1-Figure 1). These results suggested that NF-κB directly recognized the κB site and positively regulated the Bax promoter after glutamate-treatment (Figure 1D). These findings show that the p50-deficiency results in a decrease in viable RGCs due to high levels of Bax expression and caspase 3 activation, suggesting that RGCs are spontaneously to undergo apoptosis by a CaN signal cascade in the retina of p50-deficient mice.

### The effects of chemical reagents on the susceptibility to NMDA-treatment in p50-deficient mice

NMDA-receptor-mediated excitotoxicity reportedly contributes to glaucomatous neuropathy [12]. Surviving RGCs were counted at 24 h after the intravitreal injection of 10, and 5 nM, NMDAor vehicle (PBS). The treatment with 5 nM or 10 nM NMDA for 24 h markedly induced RGC death in the ganglion cell layer (GCL) in p50-deficient mice, but not in wild-type mice. Morphometric evaluation demonstrated no significant difference in the thickness of the inner plexiform layer (IPL) between the p50-deficient and wild-type mice treated with 5 nM NMDA (Appendix 1-Figure 2A). The IHC experiment clearly showed that after NMDA treatment, NF-κBp65 translocated to the nucleus in the RGCs of the wild-type as well as p50-deficient mice (Appendix 1-Figure 2B).

To determine whether Ca^2+^ signaling molecules mediate NMDA-induced neurotoxicity in p50-deficient mice, we examined the neuroprotective effects of chemical reagents, which act on the Ca^2+^-signaling pathway including CaN activation, on NMDA-induced RGC death. The p50-deficient mice at 2 months of age, showing normal RGC survival, underwent intraperitoneal pretreatments with a NMDA antagonist, MK801 or memantine; calcium blocker, lomerizine; and CaN inhibitor, tacrolimus, daily for 1 week before the injection of 5 nM NMDA (Appendix 1-Figure 3). Histological examination of the retinas at 24 h after the intravitreal injection of NMDAshowed protective effects of the chemical reagents against NMDA-induced neurotoxicity (Appendix 1-Figure 4A). Morphometric evaluation indicated marked NMDA-induced neurotoxicity in p50-deficient mice (12.3* 7.2 cells, n=12) in contrast to wild-type mice (17.9* 8.9 cells, n=10). The pre-treatments with chemical reagents markedly protected RGCs from NMDA-induced neurotoxicity (Appendix 1-Figure 4B).

In addition, RGCs were protected from NMDA-induced neurotoxicity on pre-treatment with tacrolimus at the various concentrations indicated in Appendix 1-Figure 4C. The retinal damage was reduced compared with that in mice treated with NMDAalone, even following the systemic injection of a low dose of tacrolimus.

**Figure 2:**
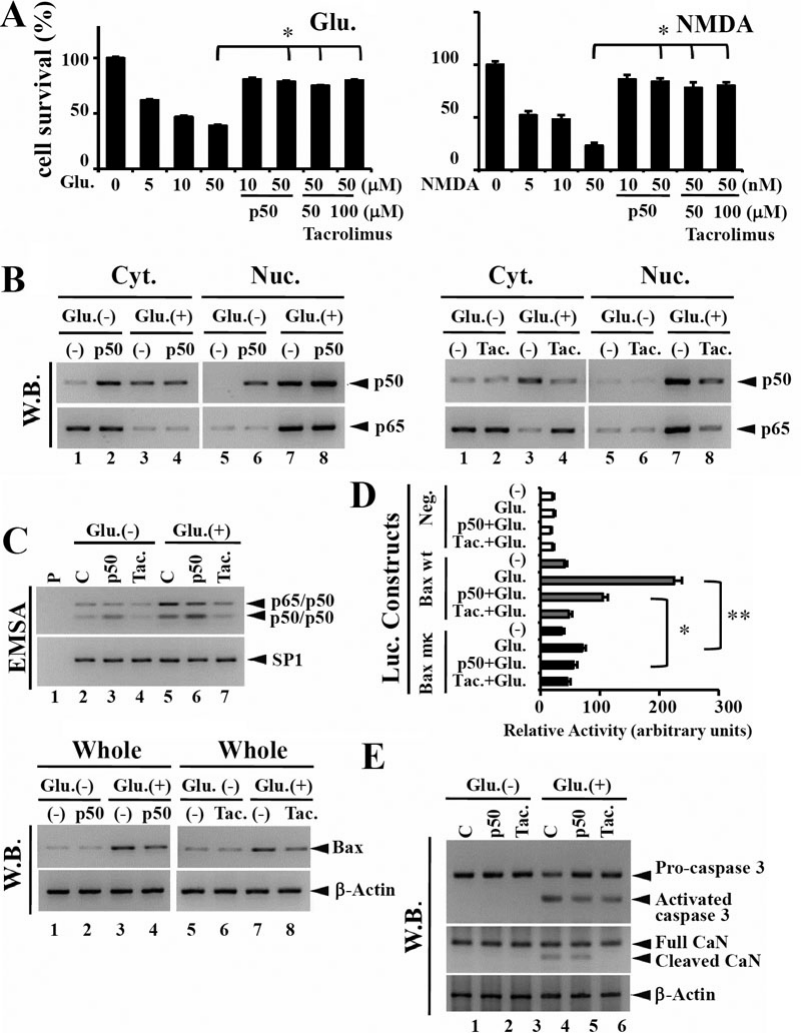
Systematic neuroprotection by systemic application of tacrolimus or p50 expression in cultured RGC-5 cells. **A**: Quantitative assessment of the protective effects of p50 expression and tacrolimus against NMDA- or glutamate-induced neurotoxicity. Cell viability was markedly decreased by the application of glutamate or NMDA. Neuroprotective effects of p50 expression or tacrolimus were observed. **B**: western blot analysis indicates the effects of p50 overexpression or tacrolimus (Tac.) pre-treatment on NF-κBp65 and p50 expression in RGC-5 cells after glutamate treatment; nucleus (Nuc.) and cytosol (Cyt.). **C**: Electrophoretic mobility shift assay (EMSA) demonstrates the effects of p50 overexpression or tacrolimus pre-treatment on NF-κB (p65/p50) activation before and after glutammate treatment. Nuclear extracts were incubated with a double-stranded oligonucleotide spanning the two B binding sites within the murine bax promoter (B sites). The arrows indicate the position of the protein-DNAcomplexes. Each lane contains the B sites probe (P) without (lane 1) or with the nuclear extracts prepared from control cells (C) RGC-5 cells (lane 2 and 5), RGC-5 cells transfected with the p50 expression vector (lane 3 and lane 6), and RGC-5 cells pre-treated with tacrolimus (Tac.; lane 4 and lane 7). Western blot analysis using whole cell extracts also indicates Bax levels under the culture conditions with p50 overexpression (left panel, lane 10, 12) or tacrolimus pre-treatment (right panel, lane 10, 12). **D**: The luciferase reporter vectors containing the wild-type or mutated B fragments of the PMBaxSacI bax promoter (−386 to −1) or the corresponding emptyluciferase reporter vector (shown in Appendix 1-Figure 1) were transiently co-transfected with or without the NF-κBp50 expression vector into RGC-5 cells. RGC-5 cells were pre-treated with tacrolimus for 24 h at 48 h after transfections with the luciferase reporter vectors. Following pre-treatment with tacrolimus, RGC-5 cells were stimulated by glutamate for 24 h, and then luciferase activities were measured. Values were normalized to those obtained with the co-transfected pSV-β-galactosidase expression vector. Each assay was performed at least three times in triplicate. Data are presented as the mean for three independent experiments (*S.D.). **E**: western blot analysis indicates the effects of p50 overexpression or tacrolimus (Tac.) pre-treatment on caspase 3 and calcineurin (CaN) activations in RGC-5 cells after glutamate treatment. Although the glutamate treatment activated caspase 3 and cleaved CaN, p50 overexpression resulted in a decrease in both active caspase 3 and cleaved CaN expression in RGC-5 cells after glutamate treatment. The pre-treatment with tacrolimus completely inhibited the glutamate-induced activation of calcineurin, and slightly decreased the level of activated caspase 3.

Glutamate-induced RGC apoptosis via calcineurin signal cascade including impaired NF-κB activation: To verify the key role of NF-κBp50 and protective effect of tacrolimus against glutamate/NMDA-induced neurotoxicity, in vitro experiments using cultured RGC-5 cell lines were performed. Glutamate/NMDA-induced neurotoxicity was clearly observed in a dose-dependent manner (Figure 2A). First, p50-overexpression markedly protected RGC-5 cells from glutamate/NMDA-induced neurotoxicity (Figure 2A). A quantitative assessment of the protective effects of tacrolimus is shown in Figure 2A. To demonstrate the biologic effect of p50-overexpression or pre-treatment with tacrolimus on NF-κB expression under glutamate- neurotoxicity, the localization of NF-κBp65 and p50 in RGC-5 cells after glutamate-stimulation was examined. WB analysis showed that glutamate markedly induced the expression and nuclear translocation of NF-κB (p65/p50 heterodimer; Figure 2B, lanes 1, 3, 5, and 7). In the case of p50-overexpression, NF-κBp50 was significantly detected in the nucleus without glutamate, and then nuclear NF-κBp50 expression markedly increased after the stimulation (Figure 2B, lanes 2, 4, 6, and 8). Experiments with EMSA revealed that after stimulation with glutamate, NF-κBp50 expression resulted in a large number of p50 homodimer complexes and a slight decrease in p65/p50 heterodimer complexes in comparison with the none transfected-cells (Figure 2C). Although Bax was strongly expressed after glutamate treatment, p50-overexpression reduced the glutamate-induced Bax expression (Figure 2C, lanes 9, 10, 11, and 12). Luciferase-reporter assays supported the WB results, suggesting that p50-overexpression significantly prevented glutamate-induced Bax expression (Figure 2C). It is possible that the binding site of the heterodimer p50-p65 could also be occupied by the homodimer p50-p50, whereupon p50-p50 may function as a repressor to regulate p50-p65's role as a transcription factor essential for neuronal response [30,31] (Appendix 1-Figure 6).

**Figure 3:**
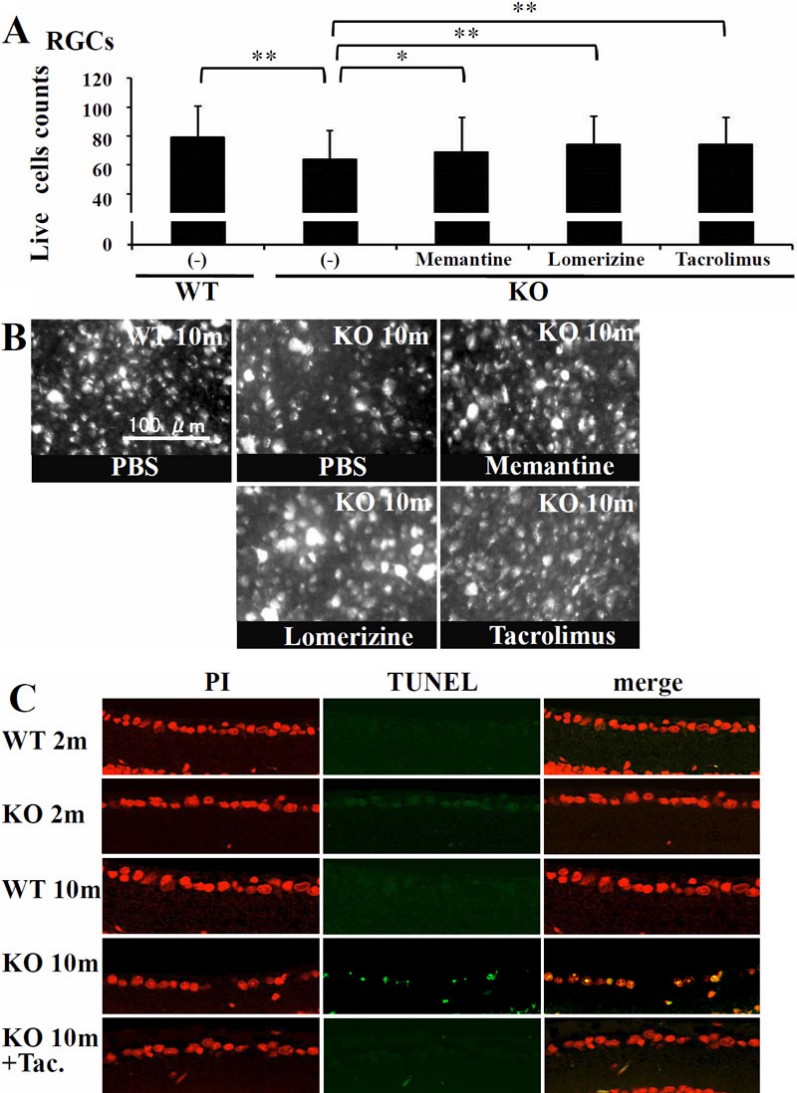
Neuroprotection by various chemical reagents in p50-deficient mice. **A**: Bar graph shows RGC survival at 8 months old p50-deficient (KO) and wild-type (WT) mice. Values represent the mean *±SD (n=each 5 11). RGC numbers of KO mice were significantly decreased (**p<0.0001). However, the chronic administration of lomerizine or tacrolimus to KO mice for 6 months resulted in an increase in surviving RGC numbers (**p<0.0001). The chronic administration of memantine to KO mice for 6 months mildly protects RGCs from neurotoxity (p=0.015). The number of surviving RGCs was counted using retrograde labeling methods. Data was analyzed by Bonferroni correction. **B**: Photographs show a high-power view of retrograde-labeled RGCs of flat mounts derived from KO and WTmice at 8 months of age. Scale bar=100 μm. **C**: TUNEL staining using the Apop Tag fluorescein labeled kit (Millipore) plus tissue sections from 2-month-old wild type (WT) mice and p50-deficient (KO) mice, and 10-month-old wild type (WT) mice and KO mice with and without chronic administration of tacrolimus. TUNEL staining was observed in the GCL of 10-month-old KO mice, but not age-matched KO mice treated with tacrolimus (Tac.) or WT mice.

Glutamate did not markedly induce NF-κBp50 expression in the cytosol or nucleus on pre-treatment with tacrolimus (Figure 2B, lanes 2, 4, 6, and 8). The pre-treatment with tacrolimus reduced the glutamate-induced translocation of NF-κB (p65/p50) from the cytosol to nucleus in RGC-5 cells (Figure 2B, lanes 7 and 8); the results obtained by EMSA strongly supported the WB results (Figure 2C). Glutamate-induced Bax expression was weakly observed in the cultures pre-treated with tacrolimus. Luciferase-reporter assays using Bax (full) promoter-Luc and Bax (ΔκB) promoter-Luc (Appendix 1-Figure 1) supported the WB results, suggesting the prevention of glutamate-induced Bax expression by pre-treatment with tacrolimus (Figure 2D). Although activated caspase 3 was clearly detected in RGC-5 cells after glutamate treatment, the overexpression of p50 or pre-treatment with tacrolimus resulted in a reduction in activated caspase 3 levels in glutamate-stimulated RGC-5 cells (Figure 3E). Cleaved CaN was observed in RGC-5 cells overexpressing p50 and control RGC-5 cells after glutamate treatment, and pre-treatment with tacrolimus inhibited CaN activation in the glutamate-stimulated RGC-5 cells (Figure 3E). As a result, the neuroprotective effect of pre-treatment with tacrolimus on glutamate-induced neurotoxicity is attributed to the prevention of CaN-induced NF-κB expression.

### The neuroprotective effect of chronic administration of chemical reagents

In the present study, we investigated the age-related survival of RGCs in p50-deficient mice, and whether the chronic administration of tacrolimus prevents the development of spontaneous optic neuropathy. We examined the neuroprotective effect of the chronic administration of chemical reagents on the development of spontaneous optic neuropathy. The intraperitoneal application of tacrolimus, and additional reagents, memantine and lomerizine, which reportedly have neuroprotective effects, daily for 8 months to p50-deficient mice was performed, and then the surviving RGCs were counted usingretrograde labeling (Appendix 1-Figure 5). The p50-deficient mice treated without the chronic administration exhibited a significant age-related decline in RGC numbers for 10 months compared with wild-type mice (Figure 3A). The p50-deficient mice treated with chemical reagents showed the following RGC counts: memantine: 69.1* 23.8 (n=9), lomerizine: 74.6* 19.4 (n=5), tacrolimus: 75.4* 18.6 (n=5; Figure 3A). Statistical analysis of the cell counts of dye-filled RGCs in flat-mounted retinas revealed significant differences between the treatments with and without chemical reagents, tacrolimus, memantine, and lomerizine (Figure 3B). The appearance of TUNEL-positive RGCs of p50-deficient mice was markedly decreased by the chronic administration of tacrolimus (Figure 3C). These in vivo experiments provided strong evidence supporting our hypothesis regarding the protective effects of the chronic administration of chemical reagents, especially tacrolimus, against age-dependent spontaneous RGC death.

**Figure 4:**
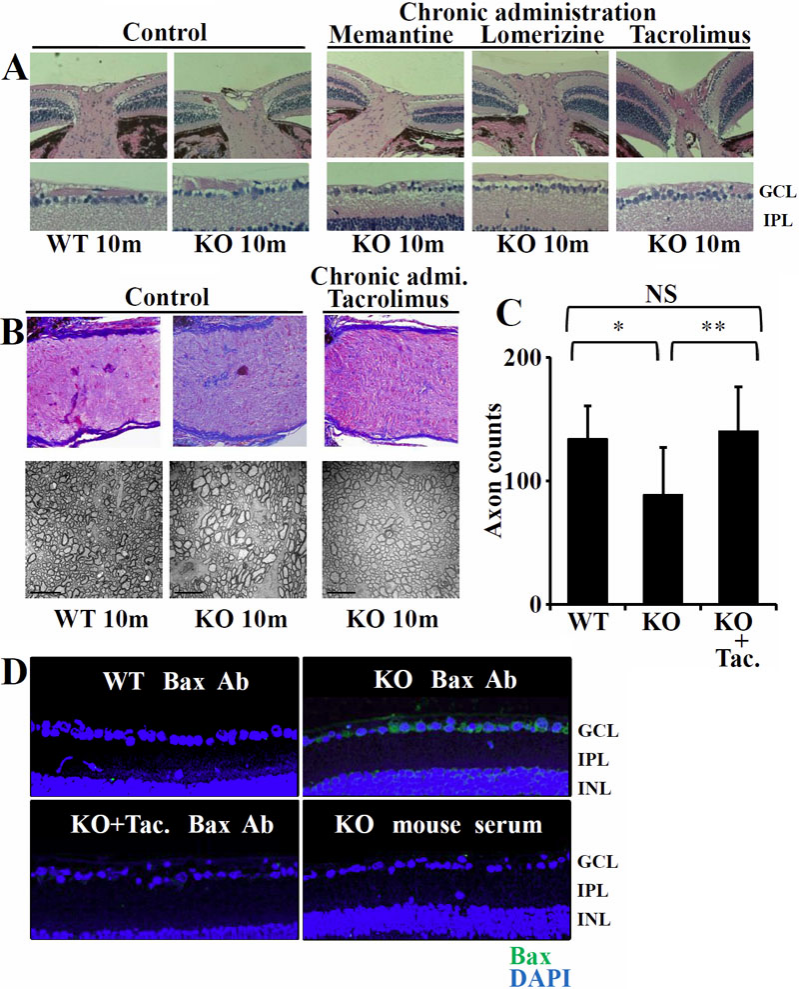
The histopathological changes and decrease in Bax expression in p50-deficient (O) mice with chronic administration of tacrolimus: **A**: Light micrographs of cross sections derived from the un-treated wild type (WT), and p50-deficient (KO) mice at 10 month of ages, and the age-matched KO mice treated by chronic administration of tacrolimus, lomerizine or memantine. Although the histopathological results show obvious excavation of the optic nerve head and RGC loss in the un-treated KO mice, no cupping is observed in the KO mice treated by chronic administration of tacrolimus, lomerizine or memantine. Serious losses in surviving RGCs are not detected in the KO mice treated with tacrolimus. Original magnification; (upper panels, low magnification 5×; lower panels, high magnification 20×). **B**: Azan staining; although the increase in collagenous fibers (blue color staining) caused by the axonal loss was clearly detected in optic nerve sections of KO mice, the chronic administration of tacrolimus prevented the increase (upper panel: original magnification; 40×). Electron microscopic examinations were performed for detection of morphological abnormalities of cross-sectioned optic nerve axons (lower panel). The KO mice exhibit an expanding area of each axon as well as decreasing number of axons in comparison with age-matched WT mice (lower panel). The area of connective tissue was significantly increased in KO mice (lower panel). However, these changes were not observed in the KO mice with the chronic administration of tacrolimus (lower panel). Scale bar=10 μm. **C**: The numbers of axons were counted from 10 fields of identical size (18 μm×18 μm). The Bar graph shows a significant decrease at 10 months of age in KO mice compared to parental mice (WT) and the KO mice treated with tacrolimus (Tac.). Data are the mean±SEM (each 5). Data were analyzed by the Mann–Whitney U-test (**p<0.0001, *p<0.001). **D**: Immunohistochemical staining with a-Bax antibody (clone 6A7) or normal mouse serum plus tissue sections from 9-month-old wild type mice (WT) and p50-deficient (KO) mice with and without chronic administration of tacrolimus. Bax expression was observed in the GCL of KO mice, but not age-matched KO mice treated with tacrolimus (Tac.) or WT mice. GCL=ganglion cell layer, IPL=inner plexiform layer. Green; Bax, Red; PI. Original magnification; 100×.

The clear excavation of the optic nerve head is observed at 10 months of age in p50-deficient mice [25]. Importantly, in the case of chemical reagent-treated mice, histological abnormalities of the optic nerve head were not detected in any mice examined (memantine: n=9, lomerizine: n=5, tacrolimus: n=5; Figure 4A). As shown in the azan staining experiments, although an increase in collagenous fibers (blue staining) caused by axonal loss was clearly detected in the optic nerve sections of p50-deficient mice, the chronic administration of tacrolimus markedly suppressed collagenous fibers (Figure 4B, upper panel). Electron microscopic analysis of a cross-sectioned optic nerve revealed that the area of each axon at 8 months of age expanded considerably more in p50-deficient mice than age-matched wild-type mice, in association with a decrease in the number of axons (Figure 4B, lower panel). However, on the chronic administration of tacrolimus, neither a decrease in the number of axons nor an increase in the area of connective tissue surrounding the axons was seen (Figure 4B, lower panel). Although the number of axons significantly decreased in p50-deficient mice compared to age-matched wild type mice, it did not decrease in the mice treated with tacrolimus (Figure 4C, n=each 5). The chronic administration of chemical reagents, especially tacrolimus, effectively protected not only RGCs but also the optic nerve from spontaneous neuropathy. The amount of Bax in RGCs of p50-deficient mice was markedly decreased by the chronic administration of tacrolimus (Figure 4D).

## Discussion

NTG shows chronic optic neuropathy and results in a loss of the visual field independently of IOP. Several population studies have suggested that NTG represents 20%–90% of all primary open angle glaucoma, with percentages seeming to vary according to race [32]. Factors, quite independent of IOP, may contribute to disease progression and recent studies have shown that glaucoma is affected by multiple genetic and environmental factors [33,34]. It should be noted that many NTG patients continue to suffer visual field loss in spite of a sufficient reduction in IOP. It is therefore important to understand the pathogenesis of NTG and establish methods of treatment.

Studies of postmortem brain tissues from patients with neurodegenerative disorders such as Alzheimer or Parkinson disease revealed increased NF-κB activity closely associated with the neurodegenerative process. NF-κB reportedly regulates apoptosis in response to stress in the nervous system, in addition to regulating apoptosis in a large variety of cells and tissues. It is difficult to perform analyses using human materials, and so animal studies complement human studies and provide important insights into human NTG. Although glutamate transporter-deficient mice also exhibit spontaneous RGC death and optic nerve degeneration without an increase in IOP, the mice don't show symptoms of chronic ailments such as in human NTG [35]. On the other hand, p50-deficient mice revealed chronic spontaneous optic neuropathy with many features resembling human NTG [25]. Because NF-κB plays such a crucial role in optic neuropathy as well as the nervous system [36-39], it is important to identify the signaling pathways leading to its activation in neurons. In our study, the constitutively active cleaved form of CaN, which has been reported to lead to apoptosis, was detected in the retina of p50-deficient mice, and then the IHC experiments showed that NF-κBp65 spontaneously translocates into the nucleolus of RGCs of p50-deficient mice (Figure 1B). Bax expression in the retina was higher in p50-deficient mice than wild type mice, and activated caspase 3 was observed in p50-deficient mice (Figure 1C). Examinations using the RGC-5 cell line also demonstrated that NF-κBp50 expression contributed greatly to the prevention of NMDA/glutamate-induced neurotoxicity and is required to protect the RGCs and optic nerve from spontaneous optic neuropathy (Figure 3). In short, p50 itself can form p50/p50 homodimers, which generally function as transcriptional repressors to regulate p50/p65's role as a transcription factor [27,40] (Appendix 1-Figure 6). Our studies suggested that the p50 deficiency leads to CaN-induced NF-κBp65 activation resulting in spontaneous RGC death based on recent research findings.

As the stimulation of glutamate receptors results in membrane depolarization which opens Ca^2+^ channels, leading to a rise in the intracellular Ca^2+^concentration, Ca^2+^ could play an important role in NF-κB activity during optic neuropathy. NF-κB's activation by various stimuli has already been shown to require Ca^2+^ for proper signal transduction [41,42]. However, the exact process involved in transducing the Ca^2+^ signal in optic neuropathy remains to be elucidated. To address this, we analyzed the neuroprotective effects of a Ca^2+^ blocker on RGCs in p50-deficient mice. A protective effect of the chemical reagents, which act on the Ca^2+^-signaling pathway, against spontaneous optic nerve neuropathy was clearly demonstrated based on the chronic administration of reagents to p50-deficient mice as well as transient experiments (Figure 2A, Figure 4A).

**Figure 5:**
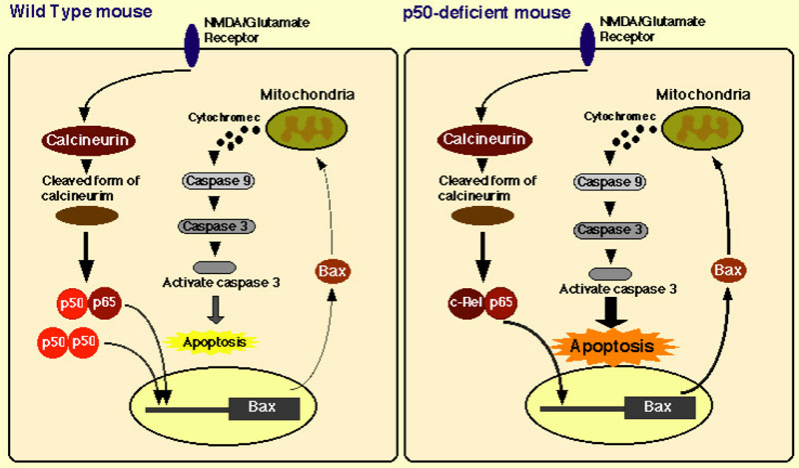
Putative biologic function of NF-κB in spontaneous optic neuropathy in NF-κBp50- deficient mouse. A calcineurin (CaN) signal cascade involving impaired NF-κB activation induces spontaneous RGC death.

CaN, a ubiquitously expressed Ca^2+^/calmodulin-dependent protein kinase II (CaMKII) -dependent protein phosphatase that is a critical component of several Ca^2+^-dependent signaling pathways, controls transcriptional regulation, cell cycle progression, and cell survival. A recent report demonstrated a marked decrease in the level of NF-κB constitutive activity when CaN was specifically inhibited, at both nuclear translocation and transactivation levels, suggesting the central importance of CaN in NF-κB activity in neurons [43]. Cleaved CaN was detected in the retina of p50-deficient mice (Figure 1C), and then the protective effect of tacrolimus against optic neuropathy was elucidated by transient experiments as well as chronic administration to p50-deficient mice (Figure 2 and Figure 3). Even though RGC-5 cells were stimulated by glutamate, the pre-treatment with tacrolimus reduced activated caspase 3 and Bax expression in RGC-5 cells. The amount of Bax in RGCs and TUNEL-positive RGCs of p50-deficient mice was markedly decreased by the chronic administration of tacrolimus (Figure 3C and Figure 4D). These research findings suggest that a CaN signal cascade involving impaired NF-Bactivation induces spontaneous RGC death (Figure 5).

Several studies have shown the potential role of the immune system, i.e., autoantibodies against ocular antigens, in the pathogenesis of glaucoma [2]. The p50-deficient mice exhibited multiple immune response defects [7]. Our previous data demonstrated the possible production of autoantibodies in the retinas of p50-deficient mice [25]. Tacrolimus is an immunosuppressive reagent whose main use is after allogenic organ transplant to reduce the activity of the patient's immune system, and so lower the risk of organ rejection. Therefore, another possibility is that tacrolimus inhibits the immune system, especially autoimmunity. To clarify the mechanism by which tacrolimus promotes RGC survival, further studies must be performed.

One important issue that remains to be resolved in neuronal physiology is how a signal that is released by neurotransmitters binding to their receptors at the synapse, and is transduced into the nucleus to activate gene transcription. Despite the importance of NF-κB to neuronal function and survival, little is known about the signaling pathways leading to its activation. In this study, we demonstrated that p50 expression is required for optic nerve cell survival, suggesting a connection between the loss of p50 expression and optic neuropathy. The mechanism whereby CaN-dependent pathways activate NF-κB is still unknown; we have chosen to utilize p50-deficient mice as well as RGC-5 cells to investigate the mechanisms by which the CaN signal cascade stimulates NF-κB-mediated neuronal cell death. The factors that act on the CaN-dependent pathway might become targets in the clinical treatment of NTG. Clinical studies have shown that there is a correlation between NTG and migraine with aura (MA): significant evidence of alinkage was found between MA phenotype and the marker D4S1647 on 4q24, where the NF-κBp50-coding gene is located [44]. Unfortunately, scientific evidence which directly shows a correlation between defective p50 expression and NTG has yet to be obtained.

In conclusion, tacrolimus protects RGCs from chronic spontaneous optic neuropathy, and then a CaN signal cascade including NF-κB activation induced chronic spontaneous optic neuropathy. Our results provide a new framework for understanding how cross-talk integrates a variety of signals in a physiologic context for the initiation of chronic optic neuropathy. These findings provide strong additional information regarding therapeutic targets for NTG.

## Appendix 1. Supplemental Information.

To access the data, click or select the words “Appendix 1.” This will initiate the download of a compressed (pdf) archive that contains the file.
